# ROS mediated MAPK signaling in abiotic and biotic stress- striking similarities and differences

**DOI:** 10.3389/fpls.2015.00769

**Published:** 2015-09-24

**Authors:** Siddhi K. Jalmi, Alok K. Sinha

**Affiliations:** National Institute of Plant Genome ResearchNew Delhi, India

**Keywords:** abiotic/biotic stress, MAPKs, protein tyrosine phosphatases, RBOH, ROS, signaling crosstalks, Sty1

## Abstract

Plants encounter a number of environmental stresses throughout their life cycles, most of which activate mitogen activated protein kinase (MAPK) pathway. The MAPKs show crosstalks at several points but the activation and the final response is known to be specific for particular stimuli that in-turn activates specific set of downstream targets. Interestingly, reactive oxygen species (ROS) is an important and common messenger produced in various environmental stresses and is known to activate many of the MAPKs. ROS activates a similar MAPK in different environmental stimuli, showing different downstream targets with different and specific responses. In animals and yeast, the mechanism behind the specific activation of MAPK by different concentration and species of ROS is elaborated, but in plants this aspect is still unclear. This review mainly focuses on the aspect of specificity of ROS mediated MAPK activation. Attempts have been made to review the involvement of ROS in abiotic stress mediated MAPK signaling and how it differentiates with that of biotic stress.

## Introduction

**FIGURE 1:**
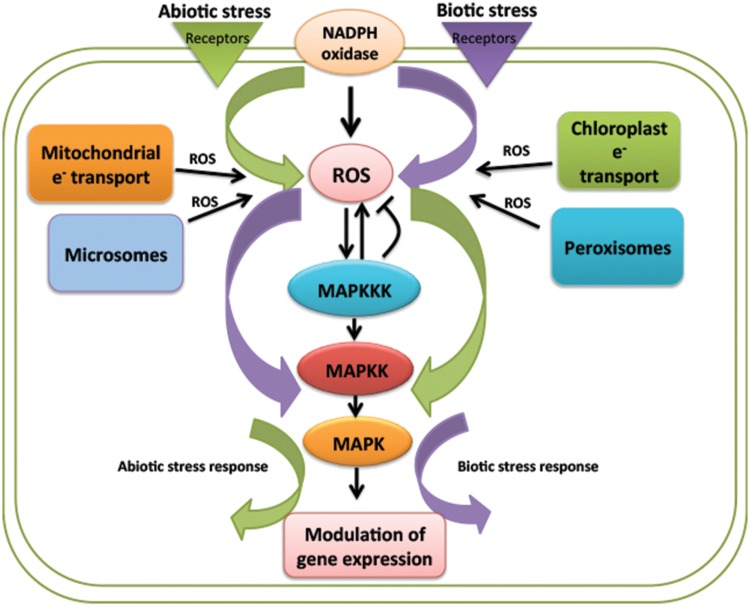
**Schematic representation of reactive oxygen species (ROS) regulation of mitogen activated protein kinases (MAPK) signaling pathway in biotic and abiotc stresses.** ROS is a common messenger produced in response to both the stress response, acting either up- or downstream of MAPK cascade. Despite being a common regulator of MAPKs signaling the response shown by plant is different in both the stresses. Purple and green color represents biotic and abiotc stress, respectively.

Plants show complex signaling network to transduce any external stimuli to the inside of the cell for an appropriate cellular arrangement giving rise to a particular response. The response is such that it helps the plant to cop up with environmental stresses that it experiences throughout its life. To exhibit a particular response, it is important for the plant to perceive the stimulus and transmit it into the nucleus of the plant cell. The perception is specifically done by cell wall receptors which then by several mechanisms activate internal signaling components. One of the most important changes that occur upon perception of external stimuli is change in redox state. Plants come across two types of stresses, abiotic and biotic. Change in redox state is a common outcome of both the stresses. This change in redox state occurs due to the production and accumulation of reactive oxygen species (ROS) in two powerhouses of plants, i.e., chloroplast and mitochondria (Apel and Hirt, 2004; Mittler et al., 2004). ROS are important secondary messengers that are poised at the core of signaling pathway in plants maintaining normal metabolic fluxes and different cellular functions (**Figure 1**). Besides chloroplast and mitochondria these are mainly produced by cell wall NADPH oxidases, peroxidases, while they are scavenged by numerous scavenging enzymes (Apel and Hirt, 2004; Nurnberger et al., 2004). The level of ROS determines whether it will be defensive or destructive molecule and its level is maintained through coordination between ROS production and turnover (Mittler et al., 2004; Miller et al., 2007). Function of ROS is also governed by its site of production, site of action and duration of action. When environmental stress becomes detrimental to the plant, it activates genetically controlled process called programmed cell death to specifically eliminate damaged tissues. In this process plants produce excess of ROS which helps in destroying stressed and damaged tissue. Signal transdcution pathways regulates the level of ROS production thereby protecting the plants from adverse effect of ROS (Bowler and Fluhr, 2000; Mittler et al., 2004).

One of the most important signaling cascades working in transmitting stress related stimuli is mitogen activated protein kinase (MAPK) cascade. MAPKs are highly conserved signaling pathway, play major role in signal transduction of diverse stress responses even in combination of many stresses. MAPK cascade consist of three tier components MAPKKKs, MAPKKs, and MAPKs carrying out phosphorylation reaction from upstream receptor to downstream target (Hamel et al., 2006). MAPKs are not only known to be activated by perception of ligand but are also activated by these ROS molecules. These phosphorylation cascades are found to work either upstream or downstream of ROS (Asai et al., 2002; **Figure 1**). MKK4-MPK3/6 module is known to play role in ROS production by acting upstream of NADPH oxidase and other way round H_2_O_2_ produced is known to activate MPK3 and MPK6 (Kovtun et al., 2000).

The manner in which plants respond to environmental stress depends on the type of stress and the outcome shown is mainly specific to particular stress. Some of the mechanisms like ROS production are common factor or outcome of both abiotic and biotic stresses whereas other mechanisms like activation of signal transduction networks, downstream activation of transcription factors and gene modulation becomes specific for specific stimuli (**Figure 1**). Question lies behind the specific activation of signaling cascade by upstream secondary messengers like ROS.

Environmental stresses encountered by plants are known to activate MAPK pathway. The MAPK activation is mostly specific but at times crosstalks are also reported in this signaling pathway (Sinha et al., 2011). Interestingly ROS which is produced by various environmental stresses is known to activate MAPKs giving a specific response. But the mechanism behind the specific activation of MAPK cascade by ROS is still unclear. This review mainly focuses on the aspect of specificity of ROS mediated MAPK activation. Attempts have been made to review the involvement of ROS in abiotic stress mediated MAPK signaling and how it delineates from that of biotic stress. In this review an update is provided on ROS regulated MAPK signaling and how it is differentially regulated by ROS produced in response to abiotic and biotic environmental stresses.

## ROS Production and its Turnover

Reactive oxygen species is being continuously produced in cell during normal cellular processes by aerobic respiration in chloroplast, mitochondria, peroxisomes, etc., ROS produced is counteracted by scavenging enzymes to maintain its level. Apart from its production from normal metabolic activities, majority of apoplastic ROS is produced by NADPH oxidase, (called as respiratory burst oxidase RBO in mammals) as first studied in mammalian ROS production. Cell wall peroxidases, germin like oxalate oxidases and amino oxidase also are involved in ROS production (Doke, 1983; Apel and Hirt, 2004; Nurnberger et al., 2004). NADPH oxidases in plants are named as Respiratory Burst Oxidase Homologs (RBOH) after their mammalian analogs. The first studied NADPH oxidase gene in plant was rice *OsrbohA* (Groom et al., 1996). Plants show different isoforms of *Rboh* genes. There exist ten *Rboh* genes in *Arabidopsis* from *AtrbohA–AtrbohJ* (Torres et al., 1998). *Rboh* genes were first identified to generate ROS in response to biotic stress. Study on mutant and antisense lines of *Rboh* genes *AtrbohD* and *AtrbohF*, gave the proof of production of oxidative burst by RBOH in pathogen infection (Torres et al., 2002). ROS generated by RBOH also impose their role in abiotic signaling and same genes are involved in ROS production in this signaling. The same Rboh isoform is able to carry out different ROS dependent function in response to different stimuli and in different cellular context (**Table 1**). The difference in outcome might exist due to complex interaction between different Rboh isoforms and with other signaling components (Foreman et al., 2003; Kwak et al., 2003; Kubo et al., 2005; Miller et al., 2009; Müller et al., 2009).

**Table 1:** Involvement of different *Rboh* genes isoforms in different environmental stresses and plant development.

*Rboh* genes	Function	Reference
*AtrbohA*	-	-
*AtrbohB*	Role in post seed ripening	Müller et al., 2009
*AtrbohC*	Root hair formation; mechanosensing	Foreman et al., 2003; Monshausen et al., 2009.
*AtrbohD*	Systemic signaling in response to diverse stimuli like pest attack, mechanical wounding, heat, cold, high light intensity and salinity. Accumulation of reactive oxygen species (ROS) in plant defense; ROS dependent ABA signaling in *Arabidopsis*.	Torres et al., 2002; Kwak et al., 2003; Miller et al., 2009; Pogány et al., 2009.
*AtrbohE*	Differentially expressed during differentiation of mesophyll cells to tracheary elements.	Kubo et al., 2005
*AtrbohF*	Accumulation of ROS in plant defense; ROS dependent ABA signaling in *Arabidopsis*.	Miller et al., 2009
*AtrbohG*	-	-
*AtrbohH*	Expressed in pollen and involved in pollen tube formation.	Potocký et al., 2007
*AtrbohI*	-	-
*AtrbohJ*	Expressed in pollen and involved in pollen tube formation.	Potocký et al., 2007

Plant response to a particular environmental stress also depends on level of ROS which is maintained by a balance between its production and turnover. This balance of ROS level is required for performing its dual role of acting as a defensive molecule in signaling pathway or a destructive molecule. There are total 152 genes involved in regulating ROS production and turnover (Mittler et al., 2004). Different antioxidants like ascorbate, tocopherol, glutathione, etc., play an important role in maintaining ROS level. Major enzymes involved in maintaining ROS homeostasis are ascorbate peroxidase (APX1), catalase (CAT1 & 2), thylakoid aperoxidase (tAPX), mitochondrial oxidase (AOX) and Cu-Zn- superoxide dismutase 2 (CSD2). Studies on mutants lacking these enzymes have revealed a strong link between biological processes, stress responses and ROS (Rizhsky et al., 2002; Pnueli et al., 2003; Miller et al., 2007).

## MAPKs Cascade Activation and ROS Generation- What Comes First?

Sensing of ROS by plant cell is done either by receptors, ROS sensitive transcription factors like heat shock factors, NPR1 or by ROS mediated inhibition of phosphatase (Mittler et al., 2004; Miller and Mittler, 2006). Once the ROS are sensed it turns on signal transduction pathway further causing differential gene expression. It can activate signal transduction pathway within the cytoplasm of cell or in the organelles where it is being produced. ROS are considered to activate signal transduction pathways in linear fashion but at times it can also work at different levels in a particular pathway. It is also likely that ROS mediated signaling pathway can act on ROS production to maintain its homeostasis in case if the ROS levels are high.

Upon perception of variety of stress stimuli MAPK cascades are activated. MAPK ultimately phosphorylate and activate several downstream targets like transcription factor, other kinases, phosphatases, and cytoskeleton associated proteins (Hamel et al., 2006; Rodriguez et al., 2010; Sinha et al., 2011). During environmental stimuli MAPKs acts on RBOH thus regulating its activity and ROS production (Asai et al., 2008). Two MAPK cascades NPK1-MEK1-NTF6 and MEK2-SIPK, known till now are found to regulate RBOH mediated oxidative burst and ROS produced is involved in mediating disease resistance (Asai et al., 2008). Recent study reported that MEKK1-MKK5-MPK6 mediates salt induced expression of iron superoxide dismutase gene further inducing ROS production (Xing et al., 2015). These studies suggest that ROS acts downstream of MAPK pathway. However, ROS an important messenger produced in various stress responses are well known to exert their effect on MAPKs, thus acting upstream of MAPKs. Upon pathogen attack ROS being produced activates *Arabidopsis* MPK3, MPK4, and MPK6. MAPK Cascade working in *Arabidopsis* in response to pathogen attack downstream of ROS is MEKK1-MKK4/5-MPK3/6 (Asai et al., 2002). Another MAPK cascade MEKK1-MKK2-MPK4/6 is known to work downstream of ROS participating in both abiotic and biotic stress signaling (Teige et al., 2004; Pitzschke et al., 2009; Furuya et al., 2014). MAPK cascades activated by ROS in particular stimuli are also known to regulate ROS production by feedback mechanism. Some studies suggest MAPK cascades to exert positive feedback regulation on ROS production. A study in maize revealed that ABA activates 46 KDa MAPK which acts downstream of H_2_O_2_ and further positively regulate RBOH for H_2_O_2_ production (Lin et al., 2009). Another cascade positively regulating ROS production is OXI1-MPK6 which is itself activated by ROS. OXI1 (Oxidative signal-induced kinase 1) is a serine/threonine MAPKKK (Asai et al., 2008). MEKK1-MKK4-MPK3/6 is known to act upstream of NADPH oxidase stimulating ROS production in pathogen attack and H_2_O_2_ produced is in turn known to activate MPK3 and MPK6 (Kovtun et al., 2000). Besides positive regulation of ROS production, MAPK cascade, NDPK2-MPK3/6 is known to negatively regulate ROS production, further giving tolerance against cold, salt, and oxidative stress (Moon et al., 2003). From these data it is clear that both ROS and MAPKs regulate each other’s activities but the mechanisms of their connections and basis of positive and negative feedback regulation still remains elusive.

## ROS Mediated Signaling Crosstalks among Various Environmental Stresses

**FIGURE 2:**
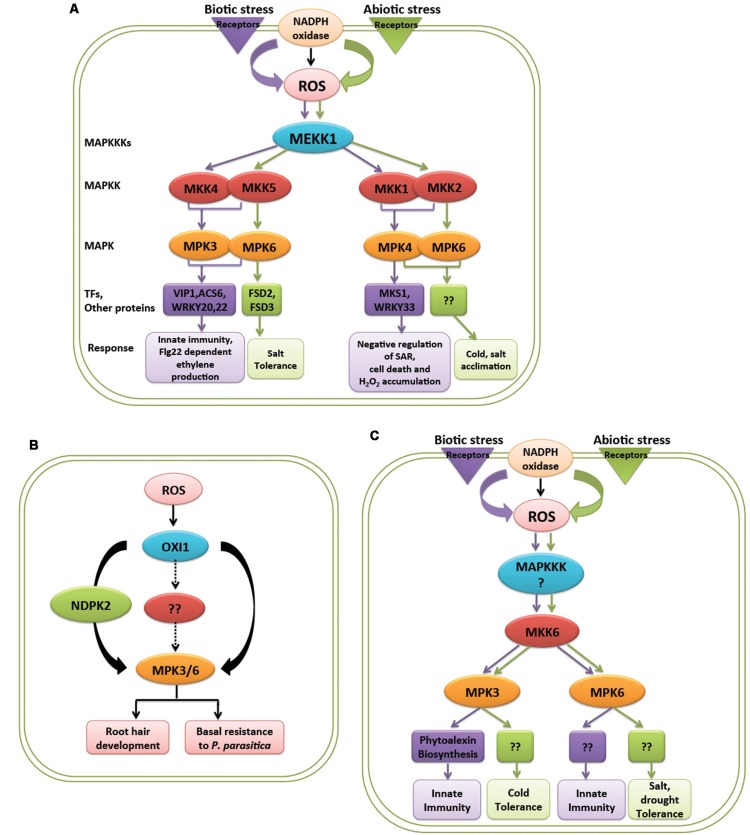
**Response of MAPK cascades activated by ROS in biotic and abiotic stresses. (A)** MEKK1 is a common MAPKKK activated by ROS produced in both biotic and abiotic stress, activates different downstream components of MAPK cascade in *Arabidopsis*. **(B)** ROS activated OXI1 mediates two different responses by activating MPK3 and MPK6 in *Arabidopsis*. **(C)** ROS generated by biotic and abiotic stress mediates different responses through activation of MKK6 and MPK3/MPK6 in rice. Purple and green color represents biotic and abiotc stress, respectively.

Mitogen activated protein kinases are important regulators of diverse cellular processes and stress responses. As an important player they show crosstalks at several points in signaling pathways in response to abiotic and biotic stresses that include ROS signaling. It is always noted that a single MAPK cascade is involved in two or more different stress responses. Also an upstream MAPK activated by a response can activate different downstream targets (Andreasson and Ellis, 2010). ROS is a common factor produced in abiotic as well as biotic stress and there are still not enough reports to clear how ROS activated MAPKs behave differently in different stress response (**Figure 2**). Below are some examples of ROS mediated activation of MAPK signaling cascades in abiotic and other environmental stresses.

In *Arabidopsis*, a MAPKKK, MEKK1 is activated upon abiotic factors like salt, cold, wound, and drought and biotic factors like bacterial and fungal elicitors (Asai et al., 2002; Teige et al., 2004; Pitzschke et al., 2009; Furuya et al., 2014; Xing et al., 2015). It is known that ROS which is being produced in these stimuli causes the activation of MEKK1. In abiotic stimuli MEKK1 activates MKK2-MPK4/6 module while in biotic stress it activates MKK4/5-MPK3/6-VIP1/ACS6 module (Asai et al., 2002; Meng and Zhang, 2013) (**Figure 2A**). Later, MEKK1-MKK1/2-MPK4 module acting upstream of MKS1/WRKY33 was also known to work in mediating pathogen related cues (Huang et al., 2000; Kong et al., 2012; **Figure 2A**). MEKK1 acting upstream of WRKY53 also showed role in plant senescence (Miao et al., 2008). ROS produced during different environmental stresses like ozone, heavy metal, biotic stress, and ABA treatment causes activation of MPK3 and MPK6 further mediating different responses (Droillard et al., 2002; Lu et al., 2002; Ahlfors et al., 2004; Liu and Zhang, 2004; Yoo et al., 2008). OXI1 is known to have different targets and show diversified activities which might suggest the crosstalk of OXI1 with other signaling pathways (Howden et al., 2011). MPK3 and MPK6 acting downstream of OXI1 mediates two different biological responses, stimulating resistance toward fungal pathogen and also play role in root development (Rentel et al., 2004; Hirt et al., 2011; Howden et al., 2011) (**Figure 2B**). Apart from OXI1, ANP1, and NDPK2 acts upstream of MPK3 and MPK6 and thus imparting tolerance to abiotic stresses like heat, cold, and salt stress (Kovtun et al., 2000).

Besides occurance of these signaling crosstalks in model plant *Arabidopsis*, it is also observed in crop plant rice. H_2_O_2_ is known to activate MPK3 and MPK6 in rice and gets activated by upstream kinase MKK6. This cascade show involvement in giving resistance to fungal pathogen as well as show tolerance to abiotic stresses, like heavy metal, salt, cold, and UV rays (Ding et al., 2009; Rao et al., 2010; Kumar and Sinha, 2013; Sheikh et al., 2013; Singh and Jwa, 2013; Wankhede et al., 2013a,b) (**Figure 2C**). The question that naturally comes to mind is what decides a same pathway to act in two different processes.

Above examples on ROS mediated crosstalks among MAPKs suggest that ROS produced in different environmental stresses mediates activation of similar MAPKs but the interaction within MAPKs and the final response toward these stresses becomes fundamentally different. At first point the differences comes from the ability of MAPKs to interact with different downstream targets. In this the scaffolding proteins also play a major role. But it also seems like ROS imparts an important role as messengers encoding total information for activating different responses.

## ROS – a Key Player in Stress Signaling but What Determines its Specificity?

The manner in which plant responds to any environmental stress depends on the type of stress and the outcome shown is mainly specific to particular stress. ROS is a common factor to both abiotic and biotic stress. Whereas, other mechanisms like activation of components in signal transduction, transcription factors becomes specific for a stress. In above mentioned studies, we saw ROS mediated activation of MAPK cascades in both biotic and abiotic stresses. The cues from different ROS molecules activating different pathways can be integrated or can activate a specific response to a single ROS molecule. MAPK pathways show convergence at several points in signaling even though activated by single messenger produced in different stresses. Beside an important role of scaffolding proteins, different ROS species also play an important role in making this difference (Torres et al., 2002; Kwak et al., 2003; Yoshioka, 2004; Miller et al., 2009). Reports suggests that the specificity of response in each stress can be due to identity of ROS species produced by different Rboh isoforms, their level, site of production and action, diffusibility and half life (Bhattacharjee, 2010; Tripathy and Oelmüller, 2012).

Plant show 10 isoforms of *RBOH* genes involved in producing different species of ROS and thus behaving differentially in various environmental cues (**Table 1**). RBOH in plants has FAD and NADPH binding motifs at C-terminal and unlike that of mammalian homolog has two Ca^+2^ binding motifs and phosphorylation target sites at N-terminal region. It is with the help of these motifs the activity of RBOH is regulated (Oda et al., 2008). The mechanism of its regulation includes phosphorylation by various signaling molecules like CDPKs, MAPKs, etc., (Lin et al., 2009). Regulation of RBOH dependent ROS production is also done with the help of amino acid residues motifs present in it. Phosphatidic acid (PA) is one of the main factors necessary for abscisic acid induced ROS production in stomatal cells. PA binding motifs present in RBOHD, i.e., Arginine residues at 149, 150, 156, and 157 are required for ROS production and closure of stomata (Zhang et al., 2009). Whereas RBOHF which is also involved in ABA dependent stomatal closure is regulated by phosphorylation of Serine 13 and Serine 174 by OPEN STOMATA 1 (OST1) (Sirichandra et al., 2009). OsRac1 involved in pathogen defense positively regulates RBOHB activity by binding to N-terminal region of RBOHB containing EF hand motifs. OsRac1 has two different forms having role in two different processes, one involved in ROS production and other in suppression of defense responses (Wong et al., 2007). This shows first step where RBOH induced ROS production is regulated in which different amino acid residues and motifs are involved in ROS production in response to different environmental responses. In addition, different RBOH homologs either single or in combination work in different stimuli giving a specific response.

Perception of different ROS species by different mechanism is still not well known and this can also explain the specific activation of downstream signal transduction by ROS in different environmental stimuli. Different locations of ROS production, different perception mechanisms and therefore different targets talks about the specificity in its response. The study on different mechanisms of action, half life and migration of different ROS species has already been carried out. The properties of most important ROS species produced in plant stress are given in **Table 2**.

**Table 2:** Properties and mode of action of ROS species on proteins.

ROS species	Source	Migration distance	Half life	Reaction with proteins residues	Reference
Hydrogen peroxide (H_2_O_2_)	NADPH oxidases and cell wall peroxidise (membrane), Chloroplast, Mitochondria, peroxisomes	1 μm	1 ms	Cystein residues	Miller et al., 2009; Bhattacharjee, 2010
Superoxide ($O_{2}^{–}$)	Membrane, chloroplast, Mitochondria	30 nm	1–4 μs	Fe-centers	Miller et al., 2009; Bhattacharjee, 2010
Hydroxyl radical (OH^-^)	Membrane, chloroplast, Mitochondria	1 nm	1 μs	Not known	Miller et al., 2009; Bhattacharjee, 2010
Singlet oxygen (O_2_)	Membrane, chloroplast, Mitochondria	30 nm	1–4 μs	Tryptophan, Histidine, Tyrosine, Methionine, Cysteine	Miller et al., 2009

**FIGURE 3:**
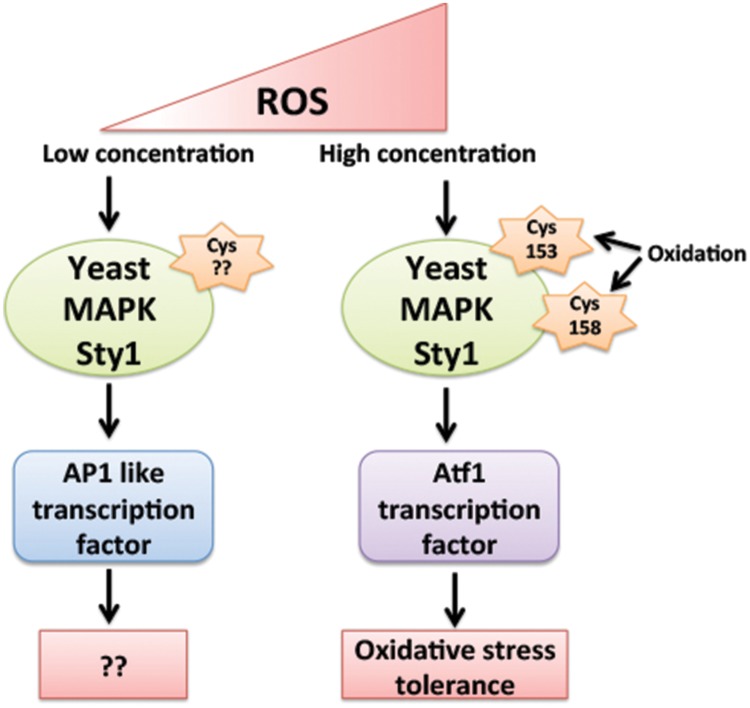
**Regulation of Yeast MAPK Sty1 (orthologs of mammalian p38 and JNK) by different levels of ROS.** Sty1 induces the expression of two different transcription factors depending on its activation by different levels of H_2_O_2_. Low levels of H_2_O_2_ activates Sty1 to induce AP1 (activator protein 1) like transcription factor, whereas higher levels of H_2_O_2_ activates Sty1 to induce Atf1 transcription factor. The difference in the activation of Sty1 substrates is due to ROS induced oxidation of different Cysteine residues of Sty1. Oxidation of Cys-153 and Cys-158 by H_2_O_2_ is essential for specific induction of Atf1 transcription factor.

The question behind the specific activation of downstream signaling components by ROS, differentially in abiotic and biotic stresses giving a specific response against a particular stress is still an enigma. The mechanism behind the specificity of MAPK activation by ROS is still elusive in plants, however, their yeast and mammalian counterparts have provided few mechanisms behind this aspect. Yeast MAPK Sty1 (Spc1, Phh1) orthologs of mammalian p38 and JNK families of MAPK play an important role in cell cycle progression and is activated in response to numerous stresses like heat, oxidative, UV, osmotic stress, and nutrient limitation (Degols and Russell, 1997). ATF transcription factor is among key substrate of Sty1 kinase. In oxidative stress conditions Sty1 not only increases phosphorylation of Atf1 but also increases its mRNA stability. Sty1 induces expression of subsets of genes in response to specific stimuli and different sets of genes are being induced by Sty1 in different concentrations of same stimuli. Low levels of H_2_O_2_ activates Sty1 to induce AP1 (activator protein 1) like transcription factor, whereas higher levels of H_2_O_2_ activates Sty1 to induce Atf1 transcription factor (Chen et al., 2008) (**Figure 3**). The difference in the downstream activation of Sty1 substrates even in response to same type of stimuli is due to H_2_O_2_ induced reversible oxidation of Cysteine residues of Sty1. Day and Veal (2010), suggested that oxidation of two Sty1 MAPKKK Cysteine residues Cys-153 and Cys-158 by H_2_O_2_ are essential for specific transcriptional activation of Atf1 transcription factor. These residues are important for hydrogen peroxide-induced gene expression and Atf1 mediated oxidative stress resistance but not for other functions of Sty1 (Day and Veal, 2010) (**Figure 3**).

Apart from the direct regulation of MAPKs by ROS, they also exert their effect indirectly through the activities of protein phosphatases and other kinases. Phosphatases are important regulators in MAPK signaling maintaining the activity of MAPKs at various points. Based on the MAPK phosphorylation sites, i.e., serine, threonine and tyrosine, phosphatases present are tyrosine phosphatases and serine/threonine phosphatases. Work carried out by scientific groups on protein tyrosine phosphatases (PTP) have suggested that reduced cysteine residue in the catalytic domain is essential for catalytic activity in plants (Gupta et al., 1998; Xu et al., 1998). A study revealed redox dependent regulation of PTP in oxidative stress. This study suggested that cysteine residues are oxidized by H_2_O_2_ in order to make PTP inactive and thus ultimately regulating MAPK signaling pathway (Blanchetot et al., 2002; Gupta and Luan, 2003). Another study in mice has put forward the possible mechanism in which age associated formation of ROS activates p38 MAPK pathway. Activation of p38 MAPK is done by ROS induced oxidation of thioredoxin and its release from the complex of ASK1 (apoptosis stimulating kinase 1). Reduced thioredoxin bound to ASK1 inhibits its activity to further activate p38 MAPK. The balance of free and bound ASK1 regulates the level of p38 MAPK components and their activity. This study suggests ROS mediated activation of p38 MAPK through unbound ASK1 and oxidation of thioredoxin (Hsieh and Papaconstantinou, 2006).

This exemplify that different types of ROS and different levels of ROS can react with different amino acid residues in protein and can give rise to different modified products, thus possibly explaining how ROS species can induce different sets of responses via the similar signaling pathway.

## Controversies about ROS Dependent MAPK Activation

Earlier studies in *Arabidopsis* suggested MPK3 and MPK6 to work upstream of AtRBOH-D ROS production and H_2_O_2_ produced was in turn known to activate MPK3 and MPK6 (Kovtun et al., 2000; Asai et al., 2008). However, a recent report suggests that AtRBOH-D dependent ROS production and MPK3/MPK6 activation are two independent events in plant immunity. It was studied using *Atrboh* mutant that flg22 triggered ROS production was blocked whereas MPK3/MPK6 activation did not get affected. It was also reported that pretreatment with SA enhance ROS production independently of MPK3/6 activation (Xu et al., 2014).

## Conclusion

Mitogen activated protein kinases are important regulators of diverse cellular processes and stress responses, showing crosstalks at several points in signaling. Single MAPK cascade is involved in two or more different stress responses. Also an upstream MAPK activated by multiple responses show different downstream targets and thus different response. ROS is a common messenger produced in abiotic as well as biotic stress activating MAPK pathways and it is still not clear in plant how ROS activated MAPKs behave differently in different stress response. Also ROS and MAPKs show feedback loop to regulate each other’s activities but the mechanisms and basis of positive and negative feedback regulation still remains elusive.

On the basis of the information available in the literature, it becomes clear that the ROS by itself has ability to regulate the downstream signaling pathway components and to impart a specific response toward a particular stress. It can activate a similar MAPK cascade in different stresses and can exert different responses accordingly. It is understood that the regulatory mechanisms of MAPKs by ROS are more elaborated in yeast and mammals, whereas in plants better understanding of the regulatory functions of ROS and MAPK cascades is required.

## Conflict of Interest Statement

The authors declare that the research was conducted in the absence of any commercial or financial relationships that could be construed as a potential conflict of interest.
